# Lessons from the recent twin earthquakes in Iran

**DOI:** 10.1371/currents.dis.ea574d0075a8e90a9cb782b368c60c36

**Published:** 2012-11-13

**Authors:** Kamyar Ghabili, Samad E J Golzari, Firooz Salehpour, Majid Khalili

**Affiliations:** Tabriz University of Medical Sciences; Tabriz University of Medical Sciences; Tabriz University of Medical Sciences; Tabriz University of Medical Sciences

## Brief Incident Report

Within eleven minutes on 11 August 2012, twin earthquakes measured 6.3 and 6.4 on the Richter scale jolted Ahar and Varzaqan in northwestern Iran, claiming 300 lives and leaving thousands of injured in rural villages.^1^ ^2^ **** The magnitude of the earthquakes was so immense that more than a hundred villages were 70-90% destroyed, while 20 being totally flattened. The primary estimations were suggestive of low number of casualties compared to the last major earthquakes that have recently happened in Iran. This could be attributed to the incident happening in the middle of the day when men had been working their fields.

The official rescue operations started after the event searching for the victims trapped under the rubble and providing emergency shelters for the survivors. However, people from the unaffected neighboring towns arrived much earlier to offer their assistance to their compatriots. Nonetheless, the death toll increased as the rescuers failed to reach the areas which were inaccessible by road and to dig the victims out of rubble in the entirely-leveled areas.

Thanks to the early establishment of triage team consisting of experienced medical staff, 961 severely injured were transferred to and underwent life-saving operations in the nearest referral hospitals in Tabriz, the capital of East Azerbaijan Province, of which 38 died.

Despite making all humanly possible attempts, there were some shortcomings and lessons to be learned for future natural disasters.

- Firstly, in the aftermath of the earthquakes, telecommunication was disrupted due to telephone network traffic overloading which in turn led to discoordination among the rescuers. This could have been avoided by providing special telecommunication equipments for the coordinators in the front line.**** 3
- Secondly, inadequacy of medical air assistance particularly during the night slowed down the speed of search and rescue.**** 4
- ****And the last, but not the least, the facilities devoted for health services should be resistant to the earthquakes as they would be considered the sole safe places for the injured and the ones in need. For instance, the two-year old hospital building in Heris, the closest city to the crisis, which was supposed to host the injured people, was destroyed in the same earthquakes.

The initial shock has now abated, though precautionary measures are still required to be taken especially considering the forthcoming cold season.

## Funding Statement

None.

## Competing Interests

The authors declare that no competing interests exist.

## Corresponding Author

Please direct correspondence to Dr. Samad EJ Golzari: dr.golzari@hotmail.com
